# Multi response optimization for enhanced xylitol production by *Debaryomyces nepalensis* in bioreactor

**DOI:** 10.1007/s13205-016-0467-x

**Published:** 2016-07-07

**Authors:** J. Sharon Mano Pappu, Sathyanarayana N. Gummadi

**Affiliations:** Applied and Industrial Microbiology Laboratory, Department of Biotechnology, Bhupat and Jyoti Mehta School of Biosciences, Indian Institute of Technology Madras, Chennai, 600 036 India

**Keywords:** Xylitol, Uniform design, Simultaneous optimization, Artificial neural network, Bioreactors

## Abstract

In this study, the optimization of different process variables—pH (4–6), aeration rate (200–550 rpm) and agitation rate (0.6–1.8 vvm) were investigated using rotating simplex method and uniform design method to enhance xylitol production from xylose by *D. nepalensis* in a batch stirred tank bioreactor. Maximum xylitol productivity (0.576 g L^−1^ h^−1^) was obtained at pH 4.0, agitation 300 rpm and aeration 1.5 vvm by rotating simplex method. Individual optimum values of pH, agitation and aeration are 4.2, 370 rpm and 1.2 vvm, respectively, for productivity, 4.3, 350 rpm and 1.0 vvm, respectively for xylitol concentration and 4.4, 360 rpm and 0.8 vvm, respectively for yield. Using generalized distance approach, the simultaneous optimal values were found to be—pH 4.3, 370 rpm and 0.9 vvm. After multi-response analysis, batch fermentation at optimal operating conditions resulted in enhanced productivity (0.76 g L^−1^ h^−1^), xylitol concentration (59.4 g L^−1^) and yield (0.58 g g^−1^) with an increase of 76.74 % of xylitol productivity.

## Introduction

Xylitol is a naturally occurring non-fermentable sugar alcohol with one third calories lesser than sucrose (Granström et al. 2007). Being a low caloric sweetener, it is used as a suitable sugar substitute for diabetic patients, parenteral nutrition (Ladefoged et al. 1982), odontological preparations (Maguire and Rugg-Gunn 2003) and also known to improve health and biomechanical properties of the bone (Mattila et al. 2002). In recent years, interest in xylitol has increased considerably, mainly due to many commercial applications in several industrial sectors like food, dental and pharmaceuticals. Increasing interest in xylitol has led to a strong demand for the product in global market. In 2013, global consumption of xylitol was estimated to be 160 thousand metric tons equating to approximately 670 million USD in value and is expected to reach 1 billion USD in 2020 (Hou-Rui 2012). To meet the world’s increasing demand, it is indispensable to produce xylitol in large scale.

The industrial production of xylitol is performed by chemical hydrogenation of xylose in the presence of metal catalysts like nickel, palladium and ruthium (Mikkola et al. 2000) at raucous operating conditions such as high temperature (80–140 °C) and 50 atm (Parajó et al. 1995). Furthermore, it also requires pure substrate (xylose) for hydrogenation, thus adding the refining cost to the total production cost. Alternatively, extraction of xylitol from natural sources is uneconomical because of its low availability (Parajó et al. 1998a). Microbial or enzymatic production of xylitol is becoming a more sustainable alternative. Biotechnological production of xylitol is gaining more interest as (1) the operating conditions are at room temperature and atmospheric pressure, (2) ease in purification and (3) relatively economical and safe process (Rodrigues et al. 2011).

Bioconversion of xylose to xylitol can be carried out by bacteria, fungi and yeast. Among the reported microbial strains, *Candida* (Barbosa et al. 1988) and *Debaryomyces* (Converti and Domínguez 2001; Converti et al. 2002) are the best known yeast species for xylitol production. Biotechnological production of xylitol is influenced by several factors which includes age and inoculum concentration, initial substrate concentration (Converti et al. 2002), pH, temperature (Converti and Domínguez 2001), aeration and agitation conditions of the fermentation process (Sampaio et al. 2008; Parajó et al. 1998b; Silva et al. 1998). pH of the medium also plays a vital role in the enhanced production of xylitol as pH affects the transport of xylose across the cell membrane (Silva et al. 2011). It has been reported that xylose to xylitol conversion by microorganisms is strongly affected by oxygen supply. Under anaerobic condition, xylose is not utilized and xylitol formation is possible only in yeast with NADH-XR (xylose reductase) activity. In the presence of excess aeration, NADH is reoxidized by respiratory chain, catalyzed by NAD^+^ dependent xylitol dehydrogenase (XDH) and xylitol is consumed for growth (Gírio et al. 1994). These results suggest that pH of the medium, aeration and agitation rates are very much crucial for enhanced xylitol production in bioreactor.

Previously, we isolated *Debaryomyces nepalensis* NCYC 3413, a halotolerant yeast strain from rotten apple, which is capable of utilizing xylose as a sole carbon source to produce xylitol (Gummadi and Kumar 2006; Kumar and Gummadi 2011a). The enzyme xylose reductase involved in the conversion of xylose to xylitol has been purified from this strain and characterized (Kumar and Gummadi 2011b). The effect of controlled pH, aeration and agitation rates on xylitol production has been studied in bioreactor (Kumdam and Gummadi 2015). To develop economical bioprocess, optimization of process conditions should be performed by considering responses such as product concentration, product yield and productivity.

To identify the effect of process parameters on the productivity and yield of xylitol, large number of experiments has to be conducted. Conventional statistical experimental methods such as the Taguchi and orthogonal experimental designs have been employed to reduce the number of experimental runs (Li et al. 2004). Fang and Wang developed a new statistical method, the uniform design (UD) of experiment, which further reduces the number of experiments when the levels of the factors are large (Fang and Lin 2003). This study deals with the optimization of different process variables—pH, aeration rate and agitation rate to enhance xylitol production in a batch stirred tank bioreactor using rotating simplex method and uniform design method. The number of experiments is low and as well as the number of the levels at which the variables studied are higher in this method when compared to other conventional statistical experimental designs (Cai et al. 2014).

An attempt has been made to determine the simultaneous optimal values of process parameters to obtain maximum yield, productivity and xylitol concentration by multi response analysis.

## Materials and methods

### Microorganism and inoculum preparation

*Debaryomyces nepalensis* NCYC 3413, isolated from rotten apple, was maintained on a solid YEPP medium containing yeast extract 10 g L^−1^, peptone 20 g L^−1^ and pectin 5 g L^−1^ at pH 7.0 and incubated at 30 °C for 24 h and stored at 4 °C. A single colony was transferred from an overnight-grown culture plate into the YEPD medium (50 ml) containing yeast extract 10 g L^−1^, peptone 20 g L^−1^ and dextrose 20 g L^−1^ and incubated for 12 h at 30 °C at 180 rpm. 8 % (v/v) seed culture was used to inoculate the fermentation medium in the stirred tank reactor.

### Fermentation medium

Semi-synthetic medium containing xylose—100 g L^−1^; (NH_4_)_2_SO_4_—3 g L^−1^; MgSO_4_—0.1 g L^−1^; K_2_HPO_4_—6 g L^−1^; Na_2_HPO_4_—3 g L^−1^; yeast extract—1 g L^−1^; CaCl_2_·2H_2_O—147 mg L^−1^; citric acid—6.9 mg L^−1^; FeCl_3_—10 mg L^−1^; MnSO_4_·H_2_O—3.4 mg L^−1^; ZnSO_4_·7H_2_O—4.3 mg L^−1^; CuSO_4_·5H_2_O—0.25 mg L^−1^; 3 N H_3_PO_4_ and 3N NaOH were used to adjust pH. All the components were autoclaved separately and mixed subsequently as described earlier (Kumdam et al. 2012).

### Batch fermentation

The batch fermentation was carried out in 2 L bioreactor (Minifors, Infors HT, Switzerland) with 1 L working volume at different combinations (Tables 1, 2) to optimize the physical parameters (pH, aeration and agitation rate). Samples were collected at regular time intervals and centrifuged at 10,000 rpm for 10 min. The supernatant was used for analysis of xylitol production and the cell pellet was used to quantify growth. Optical density was measured at *A*_600_ and cell dry weight was calculated as standardized previously for *D. nepalensis* (*A*_600_ of 1.0 corresponds to 0.34 g cell dry weight per liter culture) (Kumar and Gummadi 2011a). Fermentation runs were conducted only once since all experiments were carried out in a bioreactor with controlled conditions.

**Table 1:** Results of rotating simplex method to optimize physical parameters for xylitol production by *D. nepalensis* NCYC 3413 in batch fermentation^a^

Run no.	pH (*x*_1_)	Agitation (rpm) (*x*_2_)	Aeration rate (vvm) (*x*_3_)	Productivity (g L^−1^ h^−1^) (*Y*_1_)	Xylitol concentration (g L^−1^) (*Y*_2_)	Yield (g g^−1^) (*Y*_3_)
1	4.0	300	1.5	0.58 ± 0.003	49.9 ± 0.003	0.47 ± 0.004
2	6.0	300	0.5	0.36 ± 0.001	42.9 ± 0.001	0.47 ± 0.003
3	4.0	500	0.5	0.32 ± 0.006	26.5 ± 0.006	0.27 ± 0.005
4	6.0	500	1.5	0.47 ± 0.001	28.4 ± 0.001	0.29 ± 0.002
5	6.7	233	1.8	0.35 ± 0.003	38.0 ± 0.003	0.39 ± 0.004

^a^Experimental values are the average of duplicates with standard deviation

**Table 2:** Uniform design matrix of variables and experimental responses and predicted values of productivity, xylitol concentration and yield in batch fermentation by *D. nepalensis* ^a^

Run no.	pH (*x*_1_)	Agitation (rpm) (*x*_2_)	Aeration rate (vvm) (*x*_3_)	Productivity (g L^−1^ h^−1^) (*Y*_1_)		Xylitol concentration (g L^−1^) (*Y*_2_)		Yield (g g^−1^) (*Y*_3_)	
				Experimental	Predicted	Experimental	Predicted	Experimental	Predicted
1	6.0	550	1.0	0.23 ± 0.001	0.27	19.3 ± 0.001	14.5	0.19 ± 0.003	0.16
2	5.5	200	1.6	0.30 ± 0.002	0.27	25.1 ± 0.002	30.4	0.42 ± 0.004	0.40
3	4.0	300	1.2	0.69 ± 0.002	0.68	51.4 ± 0.002	49.8	0.54 ± 0.001	0.50
4	4.5	500	1.8	0.38 ± 0.001	0.37	32.1 ± 0.001	35.6	0.38 ± 0.002	0.35
5	7.0	250	0.8	0.49 ± 0.002	0.51	41.2 ± 0.002	38.7	0.51 ± 0.002	0.49
6	7.5	450	1.4	0.95 ± 0.001	0.93	54.5 ± 0.001	56.5	0.53 ± 0.003	0.51
7	6.5	350	2.0	0.83 ± 0.002	0.86	69.5 ± 0.002	64.7	0.61 ± 0.004	0.57
8	5.0	400	0.6	0.73 ± 0.001	0.70	39.3 ± 0.001	44.3	0.41 ± 0.002	0.40

^a^Experimental values are the average of duplicates with standard deviation

### Analytical methods

The concentration of xylose and metabolites (xylitol and glycerol) were estimated by HPLC (Jasco, Japan) equipped with refractive index detector and Aminex HPX-87H column (Bio-Rad, Richmond, USA) at 45 °C with 0.01 N H_2_SO_4_ as mobile phase at a flow rate of 0.6 ml min^−1^. The retention time of xylose was found to be 10.1 min and that of xylitol was 11.4 min. Analysis of samples were done twice and the values were used for calculating responses. Response values represented in the table were average of duplicates with standard deviation. Concentration is defined as amount of xylitol produced per liter of fermentation media. Xylitol yield and productivity are calculated as follows: yield = amount of xylitol produced (g)/amount of xylose consumed (g); productivity = xylitol concentration (g L^−1^)/fermentation time (h).

### Rotating simplex method

Aiming optimization of three physical parameters (pH, aeration rate, and agitation rate), the simplex takes the shape of a tetrahedron and the experimental design begins with a set of four experimental runs. After the initial four sets of experiments have been carried out, the experiment which gave the worst response was identified and replaced by a new combination of variables which should reflect the worst point in the response plane. However, determination of the reflection of a point of a tetrahedron in the response plane is complex, and hence a rule of thumb was applied which was found to give a satisfactory approximation of the actual reflection. The new experimental point is twice the average of the best points minus the worst point (Eq. 1) (Hendrix 1980).

1 $$R_{\text{New}} = \frac{{2\left( {R_{{{\text{B}}1}} + R_{{{\text{B}}2}} + R_{{{\text{B}}3}} } \right)}}{3} - R_{\text{w}} ,$$

where *R*_New_ is the new experimental combination, *R*_W_ is the worst point from the last four experimental runs and *R*_B1_, *R*_B2_ and *R*_B3_ are the best points from the experimental runs.

The experiment with the newly determined set of variables is then carried out and the worst response from the four remaining experiments are again identified and replaced by a new set. This iterative procedure is continued until no further improvement in response is obtained.

Setting up of high and low levels of the variables requires prior experience of the processes under study, or from values reported in the literature. Levels of the variables were chosen from the range of pH (controlled), 4.0–6.0; aeration rate, 0.5–1.5 vvm; and agitator speed, 300–500 rpm. The batch stirred tank fermentations were performed according to the design shown in Table 1. Responses–productivity (*y*_1_), xylitol concentration (*y*_2_) and yield (*y*_3_) were calculated and tabulated (Table 1). The levels of the variables for the next run were determined as per the procedure.

### Experimental design for uniform design method

A fractional factorial design named “Uniform design” (UD) was employed in experimental design of this study, which was designed by Fang and Wang from number theory (Fang et al. 2000). UD is a space filling experimental design and the basic idea of this design is to replace the complete combination of experimental parameters using relatively fewer experimental runs uniformly distributed within the parameter space (Li et al. 2004). Experimental runs were determined using the number-theoretical method and mathematically proved to be a better approximation of the complete combination of experimental parameters. The tables for arranging different experiment trials have been given in the website (http://www.sites.stat.psu.edu/~rli/uniformdesign/). UD is specifically suitable for the fermentation experiments in stirred tank bioreactor. Based on the uniform design table (Table 2) U_8_(8^3^), 8 experimental runs with 3 independent variables—pH (*x*_1_), agitation intensity (*x*_2_) and aeration rate (*x*_3_) were set for studying their effect on responses–productivity (*y*_1_), xylitol concentration (*y*_2_) and yield (*y*_3_). Responses were related to independent variables by regression analysis and were given by the following equation

2 $$y = \beta_{0} + \sum \beta_{i} x_{i} + \sum \beta_{ii} x_{i}^{2} + \sum \beta_{ij} x_{i} x_{j}$$

where *y* is the response, *β*_0_ is the intercept coefficient, *β*_*i*_ represents the linear effect and *β*_*ij*_ represents the interaction effect and β_ii_ represents the squared effect. The responses *y*_1_, *y*_2_ and *y*_3_ were treated separately to obtain the individual optimal values of the process parameters (*x*_1_, *x*_2_ and *x*_3_) using MATLAB R2009b (Mathworks, Natick, MA, USA).

### Multi response analysis

It is difficult to obtain the location of maximal points when all the responses (*y*_1_, *y*_2_ and *y*_3_) are considered simultaneously. One of the most effective techniques used in multi response analysis is the generalized distance approach (Panda et al. 1999). Let Φ_*i*_ be the optimum value of *Y*_*i*_ optimized individually over the experimental region, (*i* = 1, 2…*q*) where *q* is the number of responses considered. Location of simultaneous maxima can be found when the deviation of the multi response function is very less from the ideal optima and the condition termed as ‘near’ optimum for each predicted response can be obtained. Deviation can be compromised using the distance function which measures the distance of *Y* (*Y* = *y*_1_, *y*_2_… *y*_q_)^*T*^, considered as a point in *q*-dimensional euclidean space from Φ, the vector of individual optima. Distance function is given by *ρ*[*Y*, Φ]. The condition on *x* that minimizes the distance function over the experimental region gives the location of simultaneous maxima,

3 $$\rho \left[ {{\text{Y}}, \, \Phi } \right] \, = \left[ {\sum \left( {Y_{i} - \Phi_{i} } \right)^{2} } \right]^{{{1 \mathord{\left/ {\vphantom {1 2}} \right. \kern-0pt} 2}}}$$

where *Y*_*i*_ is the predicted *i*th response, *Y* is a matrix containing individual predicted response and Φ_*i*_ is a vector containing individual optimum value of response.

### Construction of rectangular confidence intervals

*γ*_1i_ and *γ*_2i_ boundaries of the rectangular confidence region *Dζ*, were proposed by Khuri and Conlon (1981) inequalities: *γ*_1i_ < *ζ* < *γ*_2i_. Confidence intervals were calculated as follows,

4 $$\gamma_{1i} = \, \Phi_{i} {-} \, g_{i} \left( {X_{0} ,\xi_{i} } \right)\left( {{\text{MS}}_{i} \, t_{\alpha /2,N - P} } \right)^{1/2}$$

5 $$\gamma_{2i} = \, \Phi_{i} + \, g_{i} \left( {X_{0} ,\xi_{i} } \right)\left( {{\text{MS}}_{i} \, t_{\alpha /2,N - P} } \right)^{1/2}$$

where *ξ*_*i*_ is the point at which *Y*_*i*_ attains its individual optimum Φ_*i*_, MS_*i*_ is the mean square error of the *i*th response, *N* is the number of experiments and *p* is the number of parameters in the model equation.

6 $$g_{i} \left( {X_{0} ,\xi_{i} } \right) = \left[ {Z^{T} \left( {\xi_{i} } \right)\left( {X_{0}^{T} X_{0} } \right)^{ - 1} Z\left( {\xi_{i} } \right)} \right]^{1/2}$$

where *ξ*_*i*_ is the location of variables at which *i*th response attains maximum, *Z*(*ξ*_*i*_) is the vector of location of individual maximum of *i*th response and *X*_0_ is the design matrix of order 8 × 3.

## Results and discussion

### Optimization of pH, aeration and agitation rates on xylitol production in bioreactors by rotating simplex method

The rotating simplex method is a simple and reliable technique for obtaining suitable combinations of parameters for fermentation where experiments cannot be conducted simultaneously (Panda and Naidu 2000; Xu et al. 2006). A total of five experiments were conducted to obtain the best combination of physical parameters (pH, agitation and aeration rate). During the initial four experiments, the levels of the variables were pH: 4–6; agitation rate: 300–500 rpm; aeration rate: 0.5–1.5 vvm. The above levels were set up based on the previous shake flask experiments (Kumdam et al. 2012). Initially, the four experiments have been conducted as shown in Table 1.

Xylitol production was low in run number 3 and 4, where the agitation was high. Higher agitation rate promotes growth of the organism but decreases xylitol yield. The run number 3 yielding low xylitol has been discarded and replaced by the new experimental set of variables calculated by Eq. (1). In run 5, xylitol concentration was low when compared to run 1 and 2. Improvement in production by this mechanism was unlikely, as the simplex had started moving away from the optimum combination. Therefore, the experimental values of run number 1 are considered to be the optimum. Maximum xylitol productivity (0.58 g L^−1^ h^−1^) was obtained at pH 4, agitation 300 rpm and aeration 1.5 vvm. Maximum xylitol production by *D. hansenii* around pH 4 was also reported in the work of Dominguez et al. (1997). At this optimal condition, amount of xylitol produced and product yield were 49.9 g and 0.47 g g^−1^, respectively.

### Optimization of pH, aeration and agitation rates on xylitol production in bioreactors by uniform design method

To determine the optimal value of the factors that affects xylitol production, experiments were designed using uniform design method, which includes eight experiments with eight levels for each factor (pH, aeration and agitation rates). These three variables were optimized for three responses namely productivity (*y*_1_), xylitol concentration (*y*_2_) and yield (*y*_3_) as shown in Table 2.

Analysis of the UD experiments showed that the xylitol productivity was highest (0.95 g L^−1^ h^−1^) when pH, agitation and aeration rates were at 7.5, 450 rpm and 1.4 vvm, respectively (Run # 6). Similar values of 0.83 g L^−1^ h^−1^ was obtained in run number 7 (Table 1). Low productivity (0.23 g L^−1^ h^−1^) was attained when pH, agitation and aeration rates were at 6.0, 550 rpm and 1.0 vvm, respectively (Run # 1); and similar lower values (0.3 g L^−1^ h^−1^) was obtained in run number 2 (Table 1). These results suggest that productivity is not much influenced by variations in pH of the medium but majorly depends on the aeration and agitation rates. These results are in agreement with previous reports that the conversion of xylose to xylitol largely depends on the oxygen supply to the microbial culture (Vandeska et al. 1995). It has been found that maximum xylitol concentration (69.6 g L^−1^) and product yield (0.61 g g^−1^) was obtained when pH, agitation and aeration rates were at 6.5, 350 rpm and 2.0 vvm, respectively (Run # 7). Similarly, lowest xylitol concentration (19.3 g L^−1^) and product yield (0.19 g g^−1^) was obtained when pH, agitation and aeration rates were at 6.0, 550 rpm and 1.0 vvm, respectively (Run # 1).

It was observed that xylitol yield was low (0.19 and 0.38 g g^−1^) at higher agitation rate in Run #1 (550 rpm) and in Run #4 (500 rpm) when pH was at 6.0 and 4.5, respectively. Similarly low xylitol yield (0.41 g g^−1^) was observed where agitation and aeration rates were at 400 rpm 0.6 vvm (Run #8). Xylitol yield (0.42 g g^−1^) was not improved when the agitation rates are lower (200 rpm) in run number 2. Improvement in xylitol yield from 0.54 to 0.61 g g^−1^ was noted in Run #3 and Run #7 where agitation rates are at its intermediate level 300 and 350 rpm, respectively. This can be attributed to the fact that lower agitation rate leads to oxygen limited condition, which is characterized by high energy requirement for growth and maintenance, thus affecting the xylitol production. On the other hand, higher agitation rate favors cell growth by increased oxygen availability, thus increasing the maintenance and growth requirement and causes detrimental effect on xylitol production. Responses were at its maximum when the agitation intensity was maintained at its intermediate level, which was in accordance to the results reported by Rivas et al. (2003). These results suggest that there exists strong interaction effect between the variables studied.

The data were analyzed using the statistical software Minitab 16. Regression analysis revealed the dependency of productivity, xylitol concentration and yield as a function of pH, agitation and aeration. The coefficient of the model equations were obtained by multiple regression analysis on the experimental data and are given in Eqs. (7), (8) and (9).

7 $$y_{1} = 8.704 - 0.954x_{1} - 1.090x_{2} - 5.477x_{3} + 0.064x_{1} x_{2} + 0.549x_{2} x_{3} + 0.601x_{3} x_{1}$$

8 $$y_{2} = 4.997 - 0.518x_{1} - 0.577x_{2} - 3.224x_{3} + 0.019x_{1} x_{2} + 0.333x_{2} x_{3} + 0.362x_{3} x_{1}$$

9 $$y_{3} = 3.749 - 0.334x_{1} - 0.517x_{2} - 2.153x_{3} + 0.021x_{1} x_{2} + 0.261x_{2} x_{3} + 0.216x_{3} x_{1}$$

These regression coefficients explained the effect of independent variables (*x*_1_, *x*_2_ and *x*_3_) on the responses (*y*_1_, *y*_2_ and *y*_3_). The linear coefficient term shows the direct impact of a particular factor on the response of the model equation. Coefficients of squared effects (*β*_11_, *β*_22_, *β*_33_) were statistically insignificant and the results from this study relied on the linear and interaction effects of the process variables. In Eqs. (7)–(9), coefficients *β*_23_ is in a comparable range with *β*_1_, which indicates that interaction effect of aeration and agitation plays a vital role in affecting the efficiency of xylitol production in bioreactors as mentioned earlier. The coefficient *β*_3_ implies that increase in aeration rate could cause reduction in xylitol production. In agreement to this observation, studies have shown that under aerobic conditions, xylitol yield is low (Vandeska et al. 1995). It also agrees with previous work of Preez (1994), who reported that low aeration favored whereas high aeration minimized xylitol production. Agitation rates that provides micro aerobic condition best suits xylitol production. Under this condition, the cell utilizes most of the xylose for xylitol production and the catabolic reaction was practically inactive which accounts for the accumulation of xylitol (Faria et al. 2002; Kumdam and Gummadi 2015).

It is evident from Eqs. (6)–(8) that pH has less effect on xylitol production when compared to the effect of aeration (*β*_1_ < *β*_3_) but pH should be maintained at its optimum level that well supports growth of the microbial culture and product formation. Studies on xylitol production using *D. hansenii* (Converti and Domínguez 2001) and *C. guilliermondii* (Converti et al. 2003) elucidated the existence of an optimum pH on the basis of the fact that xylose is transported across the cell membrane by a facilitated diffusion system of the proton symport type. At pH higher than optimum level, the system is limited because H^+^ transport must be performed against gradient favoring respiration. Alternatively, sub-optimal pH influences the maintenance requirement of the cell, as a result both productivity and xylitol yield decreases. Percentage correlation between experimental and model predicted were calculated and found to be high for all three responses 99.4, 96.7 and 99.6 % for productivity, xylitol concentration and yield, respectively.

Statistical test was performed for the model equations using Fischer’s statistical test for the analysis of variance (ANOVA). For best fit model, the calculated *F* value (*F*_cal_) should be greater than the tabulated *F* value (*F*_p-1, N-p_ − *F*_tab_), the instance at which null hypothesis H_0_ is rejected at α level of significance (90 %). The p value for productivity, xylitol concentration and yield were estimated to be 0.16, 0.12 and 0.11, respectively as shown in Tables 3, 4 and 5. As these* p* values are almost equal or nearer to 0.1, where α is set to 90 %, *H*_0_ is rejected at 90 % significance level and it infers that the variation accounted by the model is significantly greater than the unexplained variation.

**Table 3:** ANOVA: effect of pH, agitation and aeration on productivity of xylitol in batch fermentation by *D. nepalensis*

Source	*DF*	Seq SS	Adj MS	*F*	*P*
Regression	6	0.48	0.08	20.4	0.16
Error	1	0.00	0.00		
Total	7	0.48			

*DF* degree of freedom, *Seq SS* sequential sum of squares, *Adj MS* adjusted mean square, *F* *F* value, *P* *p* value

**Table 4:** ANOVA: effect of pH, agitation and aeration on xylitol concentration in batch fermentation by *D. nepalensis*

Source	*DF*	Seq SS	Adj MS	*F*	*P*
Regression	6	0.17	0.03	1.78	0.12
Error	1	0.02	0.02		
Total	7	0.19

**Table 5:** Effect of pH, agitation and aeration on xylitol yield (*Y*_P/S_) in batch fermentation by *D. nepalensis*

Source	*DF*	Seq SS	Adj MS	*F*	*P*
Regression	6	0.12	0.02	45.9	0.11
Error	1	0.00	0.00		
Total	7	0.12			

The regression equations were solved using MATLAB function to obtain the optimal values of the process variables. The optimal values of independent variables *x*_1_, *x*_2_ and *x*_3_ for the responses–productivity (*y*_1_), xylitol concentration (*y*_2_) and yield (*y*_3_) were determined and tabulated in Table 5. The optimal conditions of pH, aeration and agitation rates for productivity, xylitol concentration and yield were found to be 4.2, 370 rpm, 1.2 vvm, 4.3, 350 rpm and 1.0 vvm and 4.4, 360 and 0.8 vvm, respectively. Under these optimal conditions, maximum responses predicted were 0.57 g L^−1^ h^−1^, 55.0 g L^−1^ and 0.54 g g^−1^. It has also been found that predicted individual optima were almost equal to the experimental individual responses—0.59 g L^−1^ h^−1^, 56.4 g L^−1^ and 0.57 g g^−1^ as shown in Table 6.

**Table 6:** Experimental and predicted values of individual maxima, location of individual maxima and rectangular confidence intervals for optimization of xylitol production in batch fermentation by *D. nepalensis*

Response	Individual maxima		Location of individual maxima			Rectangular confidence intervals	
	Experimental	Predicted	pH	Agitation (rpm)	Aeration rate (vvm)	Lower bound	Upper bound
Productivity (g L^−1^ h^−1^) (*Y*_1_)	0.59 ± 0.003	0.57	4.2	370	1.2	0.40	1.12
Xylitol concentration (g L^−1^) (*Y*_2_)	56.4 ± 0.002	55.0	4.3	350	1.0	35.2	74.5
Yield (g g^−1^) (*Y*_3_)	0.57 ± 0.003	0.54	4.4	360	0.8	0.34	0.68

**Fig. 1:**
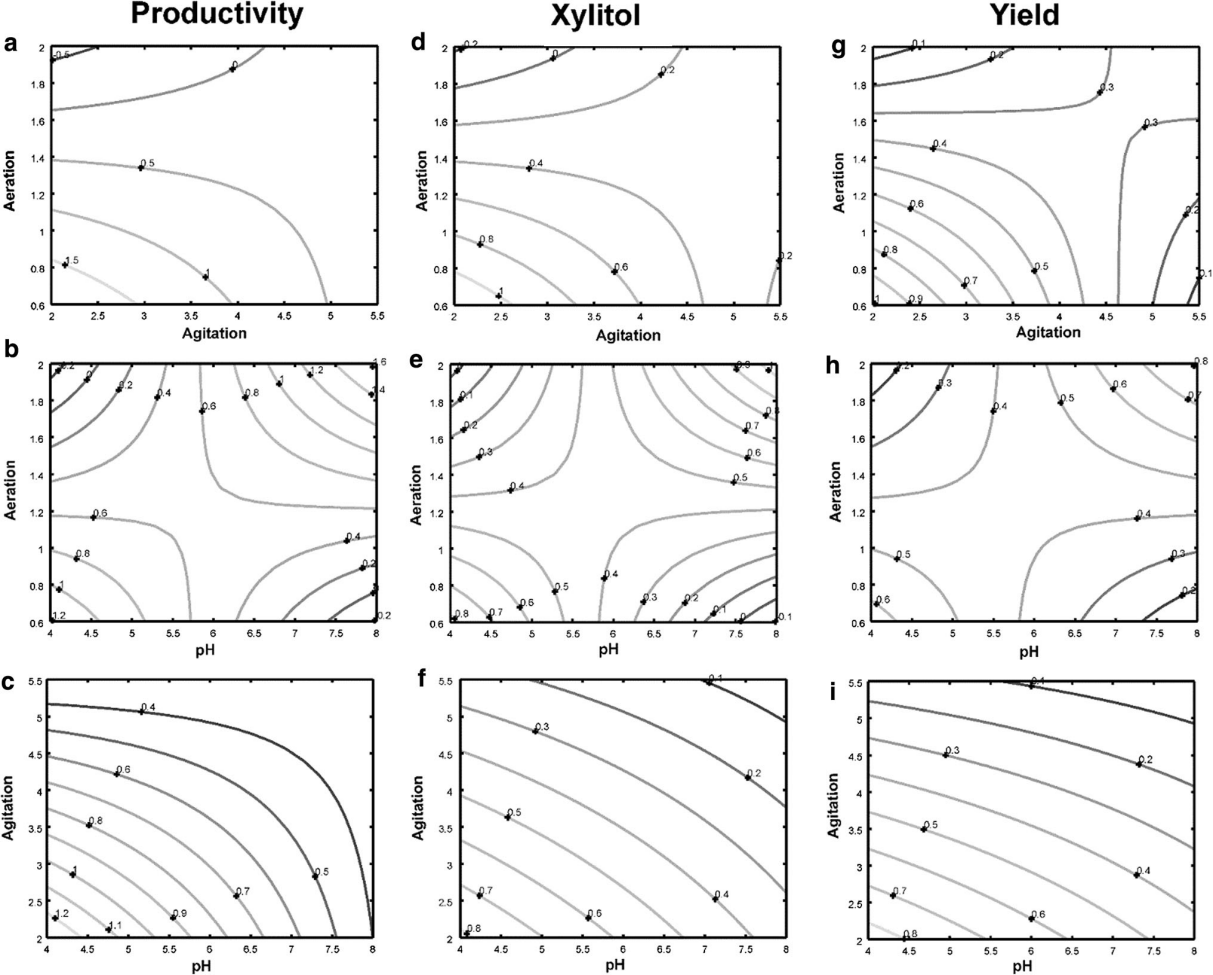
Isoresponse contour plots showing the (1) effect of aeration and agitation on productivity (**a**), xylitol concentration (**d**), yield (**g**) (at constant pH) (2) effect of aeration and pH on productivity (**b**), xylitol concentration (**e**), yield (**h**) (at constant agitation) (3) effect of agitation and pH on productivity (**c**), xylitol concentration (**f**), yield (**i**) (at constant aeration rate) in batch fermentation by *D. nepalensis*

Effect of interaction of various process parameters on the responses *y*_1_, *y*_2_ and *y*_3_ were investigated by plotting the contour plots representing the responses over changes in independent variables. As it is difficult to show the effect of three variables on responses, isoresponse contour plots were constructed by plotting the responses against any two independent variables while keeping the third independent variable at optimal value. Contour plots are the graphical representation of the regression equations. Isoresponse contour plots of productivity (*y*_1_), xylitol concentration (*y*_2_) and yield (*y*_3_) over independent variables pH (*x*_1_), agitation (*x*_2_) and aeration rate (*x*_3_) were shown in Fig. 1. Contour plots showing the effect of agitation and aeration (at constant pH) (Fig. 1a, d, g) and the plots (Fig. 1b, e, h) showing the effect of aeration and pH (at constant agitation) displays minmax or saddle behavior. In this behavior, the response reaches its maximum and moves away from it. Similar pattern of contour plots has been reported in the literature for the optimization of microbiological parameters for pectolytic enzymes production (Panda et al. 1999). Contour plots showing the effect of pH and agitation, at constant aeration rate, shows near parallel lines. These type of contour plots suggest that the interaction between the two independent variables (pH and agitation) was small. This can also be seen from the regression coefficient *β*_12_ which is small when compared to other interaction coefficients *β*_23_ and *β*_13_.

**Fig. 2:**
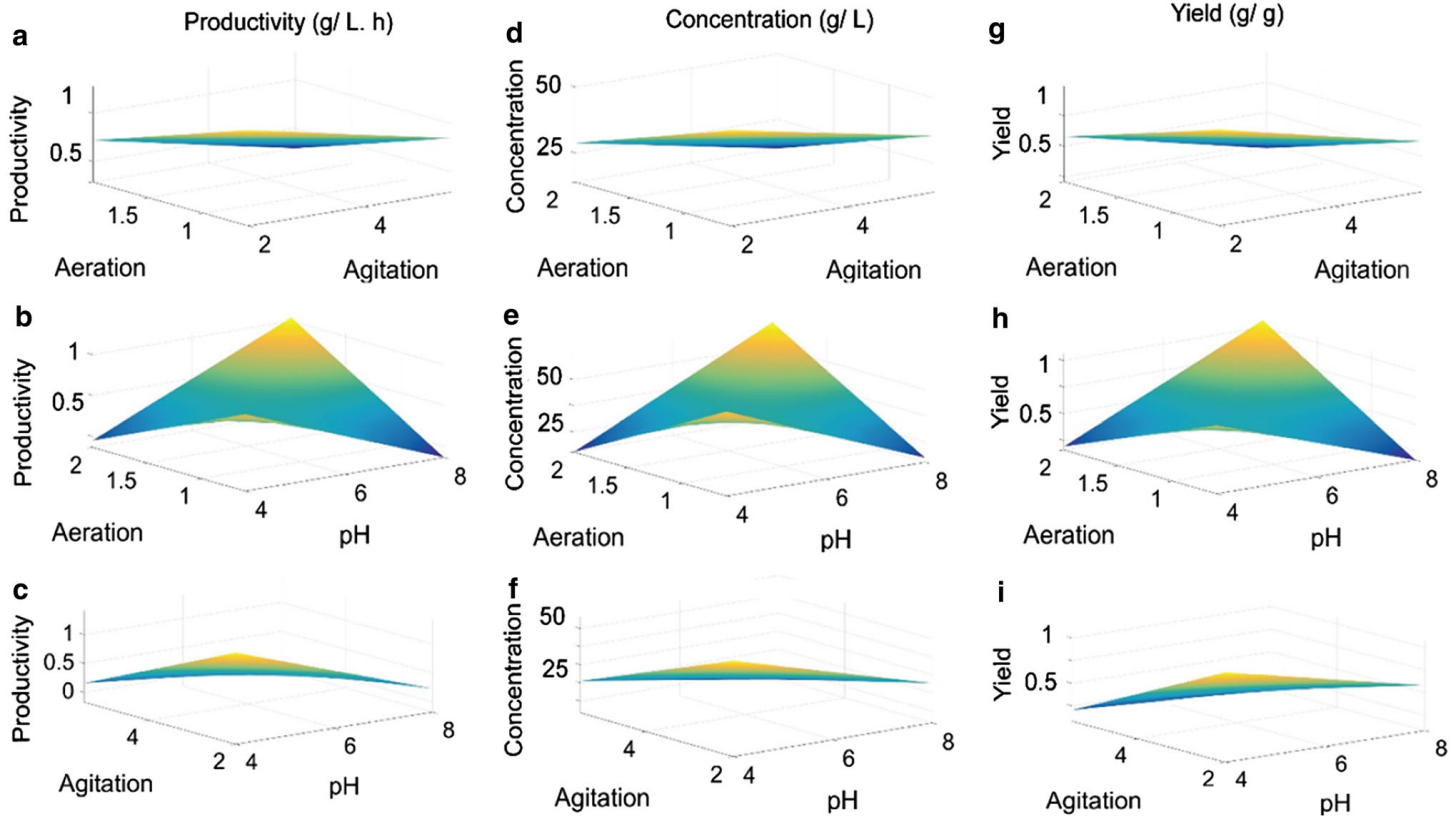
3D surface plots showing the (1) effect of aeration and agitation on productivity (**a**), xylitol concentration (**d**), yield (**g**) (at constant pH) (2) effect of aeration and pH on productivity (**b**), xylitol concentration (**e**), yield (**h**) (at constant agitation) (3) effect of agitation and pH on productivity (**c**), xylitol concentration (**f**), yield (**i**) (at constant aeration rate) in batch fermentation by *D. nepalensis* NCYC 3413

A study of the contour plots revealed that the optimal values of the independent variables lie in the following range: pH—4 to 5, agitation rate—340 to 380 rpm, aeration rate—0.8 to 1.2 vvm. The optimal values obtained from the contour plots were almost equal to the optimal value obtained by the optimization of regression Eq. (7-9). 3D surface plots exhibiting effect of aeration and agitation on productivity (Fig. 2a), xylitol concentration (Fig. 2d), yield (Fig. 2g) (at constant pH), effect of aeration and pH on productivity (Fig. 2b), xylitol concentration (Fig. 2e), yield (Fig. 2h) (at constant agitation) and effect of agitation and pH on productivity (Fig. 2c), xylitol concentration (Fig. 2f), yield (Fig. 2i) (at constant aeration rate) in batch fermentation by *D. nepalensis* NCYC 3413 were plotted in assistance to contour plots.

### Simultaneous optimization of process parameters to maximize productivity, xylitol concentration and yield in bioreactors

Location of individual maxima differs for each response as shown in Table 6. To obtain a location at which all the responses (*y*_1_, *y*_2_ and *y*_3_) attain its maximum, a multi response analysis was carried out. A generalized distance approach was used in finding out the location of simultaneous maxima. Rectangular confidence region *Dζ* was calculated using Khuri and Conlon inequalities (Eqs. 4, 5) and tabulated in Table 6. The location of simultaneous optima was obtained at which the distance function reached its minimum. Simultaneous optima and its location were calculated and tabulated in Table 7. Production and yield will be maximum when the operating conditions are at its optimal value. The result of this study confirms the influence of pH, agitation and aeration on xylitol production, productivity and yield. Experiment was performed at simultaneous optimal conditions of pH 4.3, agitation rate 370 rpm and aeration rate 0.9 vvm. The experimental xylitol productivity, concentration and yield obtained under simultaneous optimal conditions matches with the model predicted values (Table 7). Previous reactor study of xylitol production by *Debaryomyces nepalensis* NCYC 3413 reported 54 g L^−1^ of xylitol with 0.43 g L^−1^ h^−1^ productivity and 0.64 g g^−1^ yield at 0.5 vvm and 350 rpm (Kumdam and Gummadi 2015). After multi response analysis, batch fermentation at optimal operating conditions resulted in enhanced productivity (0.76 g L^−1^ h^−1^), xylitol concentration (59.4 g L^−1^) and yield (0.58 g g^−1^). Optimization of process parameters results in 76.74 and 10 % increase in productivity and xylitol concentration, respectively. 9.38 % decrease in yield after optimization can be invalidated by an increase in 76.74 % productivity. After optimization of physical parameters, productivity (0.76 g L^−1^ h^−1^) and concentration of xylitol (59.4 g L^−1^) using *Debaryomyces nepalensis* NCYC 3413 were high when compared to *Candida guilliermondii* where productivity and concentration were 0.54 g L^−1^ h^−1^ and 52 g L^−1^, respectively (Silva et al. 2006).

**Table 7:** Experimental and predicted values of simultaneous maxima, location of simultaneous maxima for optimization of xylitol production in batch fermentation by *D. nepalensis*

Response	Simultaneous maxima		Location of simultaneous maxima
	Experimental	Predicted	
Productivity (g L^−1^ h^−1^) (*Y*_1_)	0.76 ± 0.002	0.81	4.3
Xylitol concentration (g L^−1^) (*Y*_2_)	59.4 ± 0.001	55.2	370
Yield (g g^−1^) (*Y*_3_)	0.58 ± 0.002	0.49	0.9

## Conclusions

Optimizing the fermentation conditions would be more economic for enhanced production on an industrial scale. The dependency of process parameters such as pH, agitation intensity and aeration rate on productivity, xylitol concentration and yield was investigated using rotating simplex method and uniform design method. UD was proved to be a good experimental design as the number of experimental runs were reduced and specifically used in conducting bioreactor studies. Individual optimum values of pH, agitation and aeration were 4.2, 370 rpm and 1.2 vvm, respectively, for productivity, 4.3, 350 rpm and 1.0 vvm, respectively, for xylitol concentration and 4.4, 360 rpm and 0.8 vvm, respectively, for yield. The process parameters were optimized simultaneously using generalized distance approach. The simultaneous optimal values were found to be—pH 4.3, 370 rpm and 0.9 vvm. Experiments at simultaneous optimal conditions resulted in enhanced production of xylitol. In this work, analysis of experimental run was carried out by regression, which has lesser prediction accuracy when compared to neural network modelling. Optimization of parameters can also be done by artificial intelligence based methods to check further enhancement in production of xylitol.
